# Natural Th17 cells are critically regulated by functional medullary thymic microenvironments

**DOI:** 10.1016/j.jaut.2015.06.008

**Published:** 2015-09

**Authors:** William E. Jenkinson, Nicholas I. McCarthy, Emma E. Dutton, Jennifer E. Cowan, Sonia M. Parnell, Andrea J. White, Graham Anderson

**Affiliations:** MRC Centre for Immune Regulation, Institute for Biomedical Research, Medical School, University of Birmingham, Birmingham, B15 2TT, UK

**Keywords:** Thymus, Central tolerance, Thymic epithelium, nTh17, T cell, Aire, Aire, autoimmune regulator, dGuO, 2-deoxyguanosine, FTOC, fetal thymic organ culture, iNKT, invariant natural killer T, iNOS, inducible nitric oxide synthase, mTEC, medullary TEC, nTh17, natural T helper 17, nTreg, natural T regulatory, SP4, single positive CD4^+^8^−^ thymocyte, TEC, thymic epithelial cell

## Abstract

The thymic medulla is critical for the enforcement of central tolerance. In addition to deletion of auto-reactive T-cells, the thymic medulla supports the maturation of heterogeneous natural αβT-cells linked to tolerance mechanisms. Natural IL-17-secreting CD4^+^αβT-cells (nTh17) represent recently described natural αβT-cells that mature and undergo functional priming intrathymically. Despite a proposed potential to impact upon either protective or pathological inflammatory responses, the intrathymic mechanisms regulating the balance of nTh17 development are unclear. Here we compare the development of distinct natural αβT-cells in the thymus. We reveal that thymic stromal MHC class II expression and RelB-dependent medullary thymic epithelial cells (mTEC), including Aire^+^ mTEC, are an essential requirement for nTh17 development. nTh17 demonstrate a partial, non-redundant requirement for both ICOS-ligand and CD80/86 costimulation, with a dispensable role for CD80/86 expression by thymic epithelial cells. Although mTEC constitutively expressed inducible nitric oxide synthase (iNOS), a critical negative regulator of conventional Th17 differentiation, iNOS was not essential to constrain thymic nTh17. These findings highlight the critical role of the thymic medulla in the differential regulation of novel natural αβT-cell subsets, and reveal additional layers of thymic medullary regulation of T-cell driven autoimmunity and inflammation.

## Highlights

• Thymic stromal MHC class II regulates intrathymic nTh17.
• RelB-dependent medullary thymic epithelium is critical for nTh17 development.
• Aire and B7 family costimulatory ligands regulate thymic nTh17.
• Medullary thymic epithelium constitutively expresses iNOS.
• iNOS is not essential for regulation of thymic nTh17.

## 1 Introduction

The thymic medulla plays an essential, non-redundant role in the generation of central T-cell tolerance. This critical role is revealed by the manifestation of T-cell driven autoimmune disease in murine models bearing selective defects in thymic medullary microenvironments [1]. Notably, in addition to the elimination of autoreactive T-cell clones, operating through the concerted action of both medullary thymic cells and dendritic cells [2], medullary microenvironments play a pivotal role in the generation of intrathymically effector primed ‘natural’ αβT-cell subsets including natural regulatory T-cells (nTreg) and invariant natural killer cells (iNKT) [3–5]. Functionally, both nTreg and iNKT T-cell subsets possess immunoregulatory properties, being implicated in the modulation of autoimmune disease and peripheral inflammation [6,7]. In addition to imposing T-cell tolerance through negative selection, the thymic medulla therefore provides an additional layer of regulation associated with the tightly controlled developmental priming of immunomodulatory ‘natural’ αβT-cell subsets linked to both adaptive and innate-like lineages.

In addition to nTreg and iNKT, recent studies have presented evidence revealing the existence of natural IL-17 secreting αβT-cells (nTh17) within both thymic and peripheral tissues [8,9]. The precise functional contribution of nTh17 to protective immunity remains to be fully determined, although experimental evidence has proposed that nTh17 may contribute to early phase responses in murine models of respiratory challenge and oral yeast infection [10,11]. Interestingly, in parallel with peripherally induced Th17 cells, the potential protective capacity of nTh17 is counterbalanced by a proposed contribution to autoimmune disease and experimentally induced inflammatory pathology [9,12]. The requirement for tight regulation of Th17 differentiation, including nTh17, therefore represents an important mechanism impacting upon the occurrence of inflammatory disease. This point is highlighted by recent studies demonstrating that nitric oxide, derived from inducible nitric oxide synthase (iNOS) expressed by activated T-cells, constrains Th17 development and thereby suppresses IL-17 driven inflammatory disease [13].

Notably, although nTh17 share several defining characteristics of inducible Th17, including retinoic acid-related orphan receptor γt (RORγt) expression and TCR-driven IL-17 and IL-22 production, nTh17 are proposed to be distinct from peripherally induced Th17 via their innate-like capacity to produce IL-17A and IL-22 in response to TCR-independent TLR-driven cytokine stimulation. Interestingly, although nTh17 exhibit some innate-like characteristics, sequencing of the nTh17 TCR repertoire has been reported to reveal a high degree of diversity [10].

In a parallel fashion to the close developmental commonalities shared by peripherally induced Th17 and Treg, this relationship extends to nTh17 and nTreg within thymic microenvironments. In support of this, nTh17 are intrathymically selected based upon agonist stimulation by self-antigen in a manner akin to that proposed to contribute to nTreg selection, a characteristic additionally shared by iNKT although mediated by different selecting cells and ligands [8,14,15]. Given the dichotomous potential for nTreg and nTh17 to either suppress or actively drive inflammatory pathology, the intrathymic mechanisms that regulate the balance of natural αβT-cell subsets including nTh17 and nTreg, represent an important outstanding issue that remains controversial [16].

In this study, we demonstrate that thymic nTh17 are critically dependent upon MHC class II expression by thymic stroma, and moreover that intact medullary epithelial compartments are essential for nTh17 cells. Expression of the Autoimmune regulator (Aire) and both CD80/86 and ICOS-ligand co-stimulatory molecules regulates nTh17 development, although selective provision of CD80/86 by radio-resistant thymic stroma is dispensable. Finally, although mTEC compartments constitutively expressed iNOS, in contrast to the reported negative regulation of inducible Th17 via nitric oxide, absence of iNOS did not impact upon the generation of nTh17 within the thymus. Collectively, these data define differential pathways of nTh17 regulation and highlight the critical role of the thymic medulla in the regulation of nTh17 homeostasis.

## 2 Materials and methods

### 2.1 Mice

*Aire*^−/−^[17], *CD1d*^−/−^[18], *CD80/86*^−/−^[19], *Icosl*^−/−^[20], MHC class II^−/−^[21], *RelB*^−/−^[22], *Nos2*^−/−^[23] and Wildtype (WT) Balb/c, CD45.2^+^ C57BL/6 and congenic CD45.1^+^ C57BL/6 were bred and identically maintained at the Biomedical Services Unit, University of Birmingham in accordance with United Kingdom Home Office Regulations and local animal welfare and ethical approval. Embryonic mice were generated by timed mating. Detection of vaginal plug was designated as embryonic day (E) 0.

### 2.2 Antibodies, flow cytometry and cell sorting

Mechanically isolated thymocyte suspensions were stained with the following antibodies: anti-CCR6 (Biolegend, clone 29-2L17), anti-CCR7 (eBioscience, clone 4B12), anti-CCR9 (eBioscience, clone CW-1.2), anti-CD3ε (eBioscience, clone 145-2C11), anti-CD4 (eBioscience, clone GK1.5), anti-CD8 (BDbioscience, clone 53-6.7), anti-CD24 (eBioscience, clone M1/69), anti-CD44 (eBioscience, clone IM7), anti-CD45 (eBioscience, clone 30-F11), anti-CD45.1 (eBioscience, clone A20), anti-CD69 (eBioscience, clone H1.2F3), anti-CD80 (Biolegend, clone 16-10A1), anti-EpCAM (Biolegend, clone G8.8), anti-IA/IE (Biolegend, clone 114.15.2), anti-iNOS (eBioscience, clone XNFT), anti-Ly51 (Biolegend, clone 6C3), anti-Qa2 (eBioscience, clone 69H1-9-9), anti-TCRγδ (Biolegend, clone GL3), anti-TCRβ (eBioscience, clone H57-597), anti-NK1.1 (eBioscience, clone PK136). mCD1d-PBS57 tetramer reagents were kindly provided by the National Institutes of Health Tetramer Core Facility. For intracellular staining, cells were fixed and permeabilized using eBioscience Foxp3 Staining Buffer Set (eBioscience) according to manufacturers instructions and cells were incubated with anti-IL-17A (Biolegend, clone TC11-18H10.1), anti-Foxp3 (eBioscience, clone FJK-16s) and anti-RORγt (eBioscience, clone AFKJS-9). Flow cytometry data was acquired using a BD Fortessa running FACSDiva6.2 software, and subsequently analyzed using FlowJo software (Treestar).

### 2.3 Cell stimulation

For intracellular cytokine labeling, suspensions of thymocytes were stimulated *in vitro* with Ionomycin (1.5 μM) and phorbol myristate acetate (PMA) (50 μg/ml) with the addition of Brefeldin A (10 μg/ml) (all Sigma–Aldrich) for 4 h prior to antibody labeling.

### 2.4 Fetal thymus organ culture and thymus transplantation

Fetal thymic organ cultures used for the detection of intrathymic Th17 were performed via the incubation of E16 WT Balb/c fetal thymi in organ culture for seven days [24]. For fetal thymus transplantation experiments, E15 *RelB*^−/−^ and WT C57BL/6 fetal thymi were cultured for seven days in the presence of 1.35 mM 2-deoxyguanosine (dGuO) and subsequently transplanted under the renal capsule of 8-week old WT host mice as reported [25].

### 2.5 Bone marrow irradiation chimeras

Bone marrow cells were isolated from the femurs and tibias of WT CD45.1^+^ adult mice. T-cells were depleted from bone marrow suspensions using PE-conjugated anti-CD3e and anti-PE microbeads (Miltenyi Biotec). CD45.2^+^ WT, *Cd80/86*^−/−^ and MHC class II^−/−^ hosts were lethally irradiated with two split doses of 450 rad and subsequently reconstituted with 5 × 10^6^ CD3ε-depleted CD45.1^+^ WT donor bone marrow.

### 2.6 Statistical analysis

Statistical analysis was performed with Prism software (GraphPad) using a non-parametric Mann–Whitney *U* test or a non-parametric Kruskal–Wallis one-way analysis of variance with Dunn's post-test for comparison of more than one group. *p* values of <0.05 were designated to be statistically significant.

## 3 Results

### 3.1 Discrimination of natural αβT-cell subsets within murine thymus

**Fig. 1:**
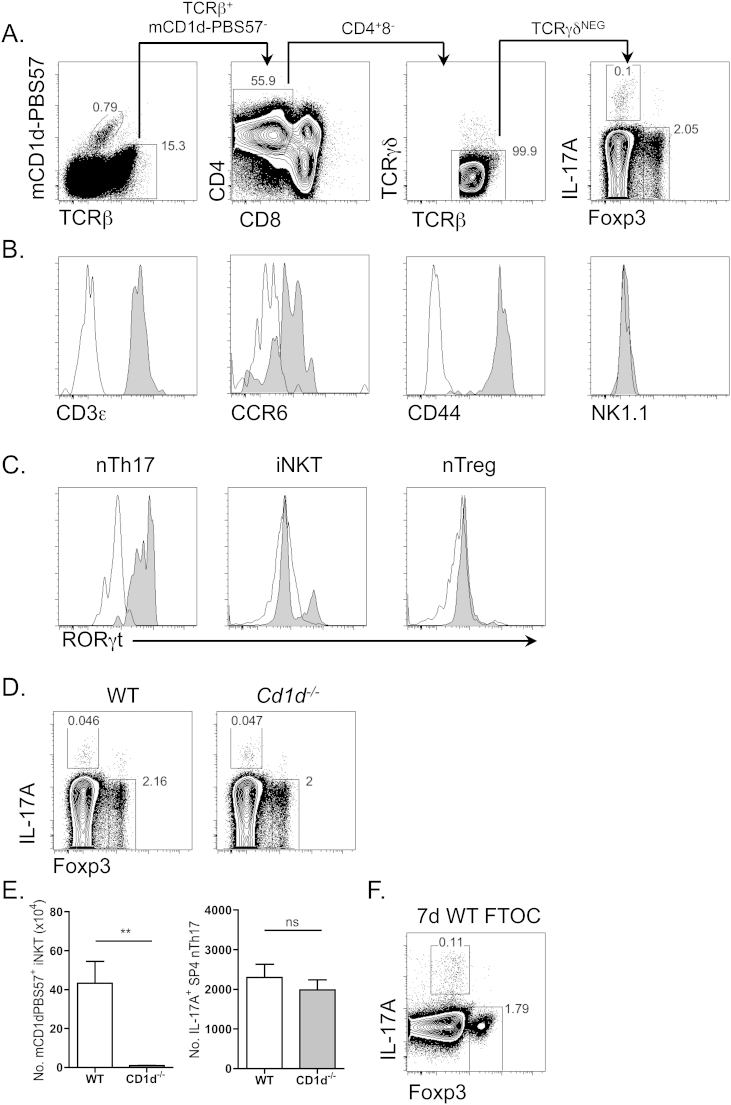
**Discrimination of distinct, thymic resident natural αβTCR subsets**. (A) Gating strategy for flow cytometric analysis of TCRβ^+^mCD1d-PBS57^+^ iNKT cells, TCRβ^+^mCD1d-PBS57^−^CD4^+^CD8^−^TCRγδ^−^Foxp3^+^ nTreg and TCRβ^+^mCD1d-PBS57^−^CD4^+^CD8^−^TCRγδ^−^IL-17A^+^ nTh17 cells following isolation of adult wildtype (WT) murine thymocytes stimulated *in vitro* with PMA and Ionomycin. (B) Histogram plots depict expression of the stated cell surface protein in nTh17 cells (grey-filled histogram) by flow cytometry. Cells gated as above, data representative of two independent experiments. Isotype staining controls represented by black open histograms. (C) RORγt expression (grey-filled histogram) in nTh17, nTreg and iNKT cells by flow cytometry. Cells gated as above, data representative of at least two independent experiments. Isotype staining controls represented by black open histograms. (D, E) Thymocytes from adult WT and CD1d^−/−^ mice were stimulated and analyzed by flow cytometry as in A. Panel (D) depicts representative staining within TCRβ^+^mCD1d-PBS57^−^CD4^+^CD8^−^TCRγδ^−^ gated cells. The absolute number of iNKT and nTh17 are demonstrated in WT (open bars) and CD1d^−/−^ (grey bars) adult thymi (E). Error bars represent SEM. Using a Mann Whitney U test, ns (not significant), **p < 0.01. Data is from 2 independent experiments, with a total of n ≥ 5 mice per group. (F) Thymocytes isolated from E16 WT Balb/c fetal thymus organ after 7 days culture were stimulated and analyzed by flow cytometry. Representative plot gated on TCRβ^+^mCD1d-PBS57^−^CD4^+^CD8^−^TCRγδ^−^ cells. Data representative of two independent experiments, with n > 20 pooled fetal thymic lobes per experiment.

Within thymic microenvironments, the detection of natural Th17 cells is potentially confounded by the presence of multiple T-cell and innate-like lymphoid cells that possess the shared capacity to produce IL-17, including γδT-cell, iNKT and group 3 innate lymphoid cell (ILC) subsets [26]. Following Ionomycin and PMA stimulation, a strict gating strategy was adopted to selectively identify αβTCR^+^ natural T-cells including; iNKT cells (mCD1d-PBS57 tetramer^+^), IL-17 producing nTh17 cells (TCRβ^+^PBS57^−^TCRγδ^−^CD4^+^CD8^−^IL-17^+^) and Foxp3^+^ nTreg (TCRβ^+^PBS57^−^TCRγδ^−^CD4^+^CD8^−^Foxp3^+^) (Fig. 1A). In order to confirm that nTh17 were T-lineage cells, and not due to experimental overlap with thymic resident non-T-cell innate populations, including CD3ε^−^ group 3 ILC subsets that can potentially produce IL-17 and IL-22 [27], we confirmed that in addition to TCRβ expression, nTh17 were CD3ε^+^ (Fig. 1B and Supplementary Fig. 1).

nTh17 detected within adult murine thymus demonstrated an activated/memory phenotype, being CCR6^+^CD44^HI^, and in contrast to the majority of thymic iNKT, nTh17 were characterized by an absence of NK1.1 expression (Fig. 1B and Supplementary Fig. 1). Analysis of the transcription factor RORγt, revealed expression within nTh17 populations (Fig. 1C). Given that intrathymic iNKT17 subsets share a potentially similar spectrum of phenotypic and functional overlap with nTh17, including being CD44^HI^NK1.1^−^RORgt^+^ (Fig. 1B and C and Supplementary Fig. 1) [28,29] and both having the innate-like capacity to rapidly produce IL-17, including via both TCR and TLR-driven mechanisms [9,30], we sought to ensure that detection of nTh17 was again not due to experimental contamination by CD1d-dependent thymic NKT populations. As expected, analysis of thymus from adult CD1d-deficient mice revealed a clear absence of mCD1d-PBS57^+^ iNKT cells, however nTh17 were found to be present both at normal frequencies and numbers in the absence of thymic CD1d-dependent NKT fractions (Fig. 1D and E).

Given that intra-thymic nTh17 display characteristics associated with peripherally activated T-cell subsets, (CCR6^+^CD44^HI^), we next sought to definitively assess whether nTh17 truly represent intra-thymically primed populations and are not detected due to the thymic re-entry of recirculating αβT-cells that have undergone priming in response to peripheral inflammatory signals. In agreement with previous reports that nTh17 are intrathymically generated based upon analysis of levels of GFP expressed under the control of the RAG2 promotor, nTh17 were detected in fetal thymic organ cultures (FTOC), where embryonic day 16 WT Balb/c thymi were incubated for 7-day *in vitro* culture at stages prior to T-cell export from thymic tissues (Fig. 1F). Together these data support the proposal that nTh17 represent a discrete αβT-cell subset, distinct from thymic iNKT, and that generation of nTh17 can specifically occur in an intra-thymic fashion in the absence of peripheral stimulation and/or re-circulation.

### 3.2 Thymic nTh17 are dependent upon functional medullary microenvironments

**Fig. 2:**
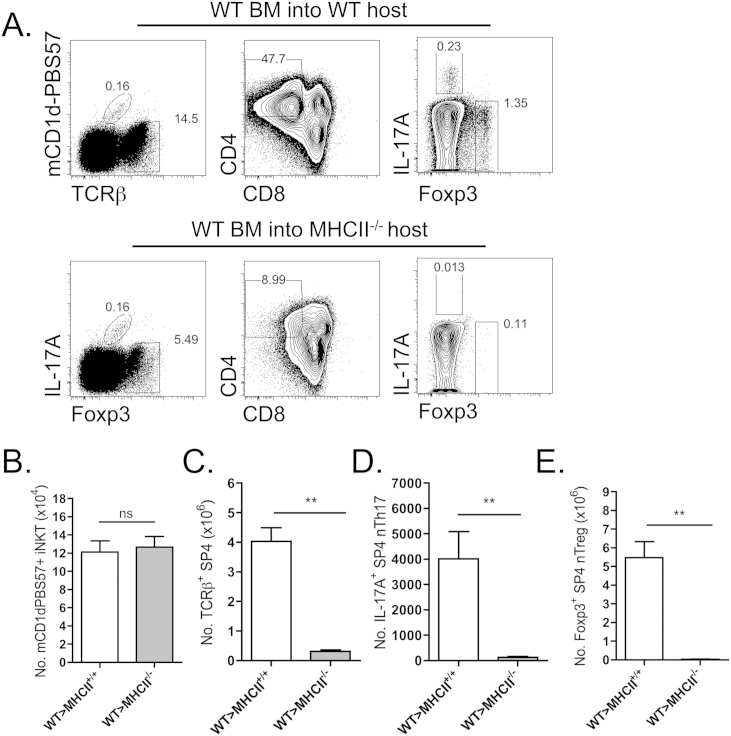
**Expression of MHC class II by radioresistant thymic stroma is essential for intrathymic nTh17**. Lethally irradiated CD45.2^+^ WT or CD45.2^+^ MHC class II^−/−^ hosts were reconstituted with congenic CD45.1^+^ CD3e^+^-depleted bone marrow to target MHC class II-deficiency to radioresistant thymic stromal elements. (A) Representative flow cytometry staining of PMA/ionomycin stimulated thymocytes, for CD45.1^+^mCD1d-PBS57^+^ iNKT cells (left panels), CD45.1^+^mCD1d-PBS57^+^TCRβ^+^CD4^+^CD8^−^ SP4 thymocytes (middle panels) and CD45.1^+^TCRβ^+^mCD1d-PBS57^−^CD4^+^CD8^−^TCRγδ^−^ gated IL-17^+^ nTh17 and Foxp3^+^ nTreg (right panels). (B) Absolute numbers of mCD1d-PBS57^+^ iNKT cells, (C) TCRβ^+^mCD1d-PBS57^−^CD4^+^CD8^−^ SP4 thymocytes, (D) TCRβ^+^mCD1d-PBS57^−^CD4^+^CD8^−^TCRγδ^−^IL-17A^+^ nTh17 cells and (E) TCRβ^+^mCD1d-PBS57^−^CD4^+^CD8^−^TCRγδ^−^Foxp3^+^ nTreg. Error bars represent SEM. Using a Mann Whitney U test, ns (not significant), **p < 0.01. Data is from 3 independent experiments, with a total of n ≥ 5 mice per group.

The development of iNKT and nTreg natural αβT-cell subsets demonstrates divergence in both selecting ligands and selecting cell types. Notably, iNKT undergo selection via CD1d:glycolipid presentation by CD4^+^8^+^ thymocytes, whereas nTreg undergo selection via self-peptide:MHC class II presented by mTEC in combination with thymic dendritic cells (DC) [31,32]. However, in contrast both iNKT and nTreg demonstrate a shared dependency upon intact medullary thymic microenvironments for their full maturation. Given that nTh17 have been proposed to share developmental commonalities with nTreg, we investigated whether nTh17 displayed a similar reliance upon MHC class II as nTreg for their selection. Analysis of MHC class II-deficient adult mice revealed an absence of nTh17 within thymic tissues (data not shown). Further, the generation of radiation chimeras to selectively target loss of MHC class II to radio-resistant thymic stromal elements (Fig. 2), revealed that in contrast to MHC class II-independent iNKT development (Fig. 2A and B), expression of MHC class II by thymic stroma was essential for both nTreg and nTh17 (Fig. 2A, D and E).

**Fig. 3:**
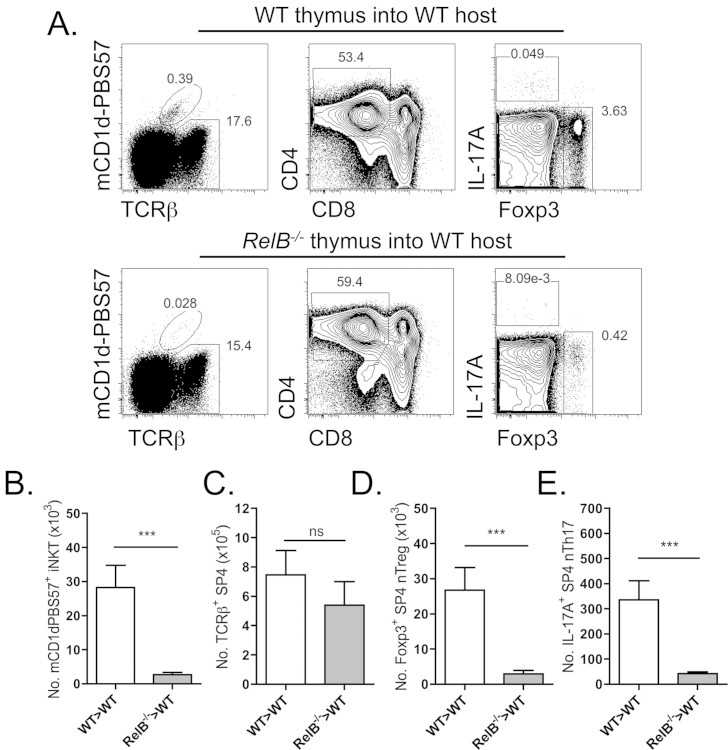
**RelB-dependent medullary thymic epithelium is essential for intrathymic nTh17**. Thymocytes isolated from WT and *Relb*^−/−^ 2-dGuO-treated embryonic thymus grafted under the renal capsule of WT adult hosts for >8 weeks were analyzed for natural αβT-cell development. (A) Representative flow cytometry staining of PMA/ionomycin stimulated thymocytes, for CD45.1^+^mCD1d-PBS57^+^ iNKT cells (left panels), CD45.1^+^mCD1d-PBS57^+^TCRβ^+^CD4^+^CD8^−^ SP4 thymocytes (middle panels) and CD45.1^+^TCRβ^+^mCD1d-PBS57^−^CD4^+^CD8^−^TCRγδ^−^ gated IL-17^+^ nTh17 and Foxp3^+^ nTreg (right panels). (B) Absolute numbers of mCD1d-PBS57^+^ iNKT cells, (C) TCRβ^+^mCD1d-PBS57^−^CD4^+^CD8^−^ SP4 thymocytes, (D) TCRβ^+^mCD1d-PBS57^−^CD4^+^CD8^−^TCRγδ^−^IL-17A^+^ nTh17 cells and (E) TCRβ^+^mCD1d-PBS57^−^CD4^+^CD8^−^TCRγδ^−^Foxp3^+^ nTreg isolated from WT (open bars) and *Relb*^−/−^ (grey bars) thymus grafts. Error bars represent SEM. Using a Mann Whitney U test, ns (not significant), ***p  < 0.001. Data is from 2 independent experiments, with a total of n ≥ 8 thymus grafts analyzed per group.

Previous studies have highlighted the role of an intact thymic medulla in the regulation of iNKT and nTreg maturation, including a specific requirement for RelB-dependent mTEC [3,33]. In order to determine whether RelB-dependent mTEC regulate nTh17 development, we took advantage of an ectopic thymus transplantation model, whereby surgical transfer of dGuO-treated wildtype or RelB-deficient fetal thymi under the renal capsule of host adult wildtype mice facilitates the study of T-cell development within RelB-dependent mTEC-deficient grafts (Fig. 3), as previously described [3]. Importantly, this thymus transplantation model facilitates the analysis of T-cell development in thymic microenvironments selectively lacking RelB-dependent mTEC without the compounding effects of peripheral inflammation that otherwise occurs in RelB knockout mice [22]. Critically, we observed a dramatic decrease in both the proportion and number of all three natural αβTCR iNKT, nTreg and nTh17 populations despite non-significant changes within the number and relative frequency of conventional αβTCR SP4 thymocytes (Fig. 3A–E). Together these data imply that a functional thymic medulla is essential for natural αβT-cell development, including nTh17, and further that the expression of MHC class II by cortical thymic epithelium within RelB-dependent mTEC-deficient thymic grafts is insufficient for nTh17 development.

### 3.3 Aire and CD28 superfamily co-stimulation regulate thymic nTh17

**Fig. 4:**
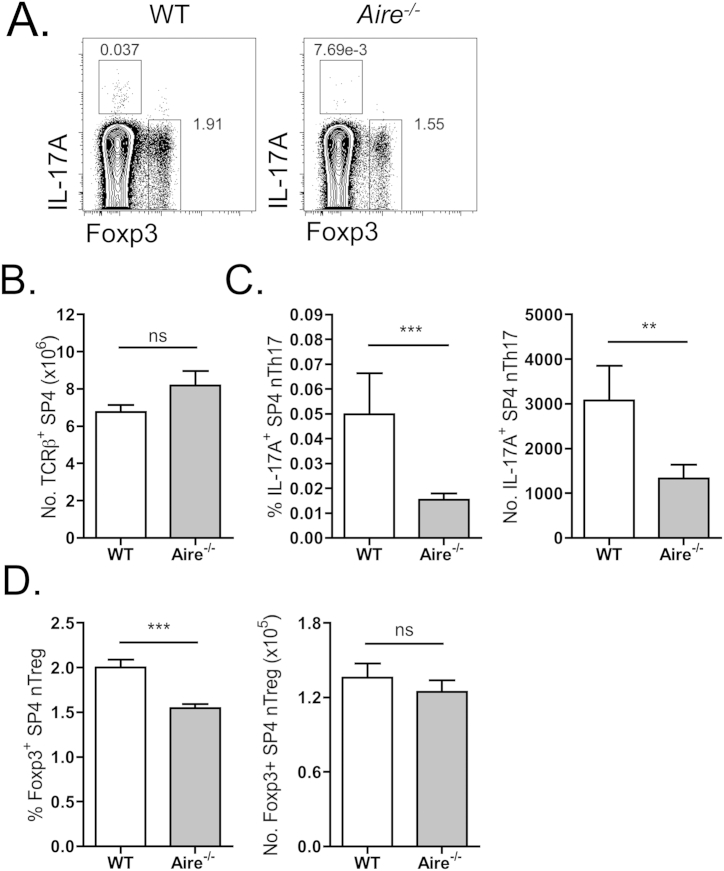
**Aire expression regulates thymic nTh17**. (A) Representative flow cytometric staining of PMA/Ionomycin stimulated thymocytes isolated from WT and *Aire*^−/−^ adult murine thymus. Representative plots gated on TCRβ^+^mCD1d-PBS57^−^CD4^+^CD8^−^TCRγδ^−^ cells. Absolute numbers of TCRβ^+^mCD1d-PBS57^−^CD4^+^CD8^−^ SP4 thymocytes (B), the frequency and absolute number of TCRβ^+^mCD1d-PBS57^−^CD4^+^CD8^−^TCRγδ^−^IL-17A^+^ nTh17 cells (C), and TCRβ^+^mCD1d-PBS57^−^CD4^+^CD8^−^TCRγδ^−^Foxp3^+^ nTreg (D) are depicted. WT (open bars) and *Aire*^−/−^ (grey bars). Error bars represent SEM. Using a Mann Whitney U test, ns (not significant), **p < 0.01, ***p < 0.001. Data is from 4 independent experiments, with n = 9 mice per group.

Due to the above results highlighting that thymic nTh17 demonstrate an essential requirement for intact medullary epithelial microenvironments, we investigated specific mTEC-associated mechanisms responsible for controlling the balance of nTh17 development. Analysis of adult mice lacking expression of Aire, the transcriptional regulator linked to the intrathymic expression of peripheral tissue-associated antigens and associated enforcement of central T-cell tolerance [34], revealed a significant reduction in the percentage and total number of thymic nTh17 (Fig. 4C). Importantly, the reduction observed within nTh17 thymocyte fractions was not simply due to a gross decrease in mature thymocyte subsets, as conventional TCRβ^+^ SP4 thymocytes were present in normal numbers in Aire-deficient mice compared to wildtype counterparts (Fig. 4B). In addition, and in agreement with previous reports [35–37], analysis of Foxp3^+^ nTreg revealed a significantly reduced proportion amongst TCRβ^+^ SP4 thymocytes, although total nTreg numbers were not found to be significantly reduced in this system (Fig. 4D).

**Fig. 5:**
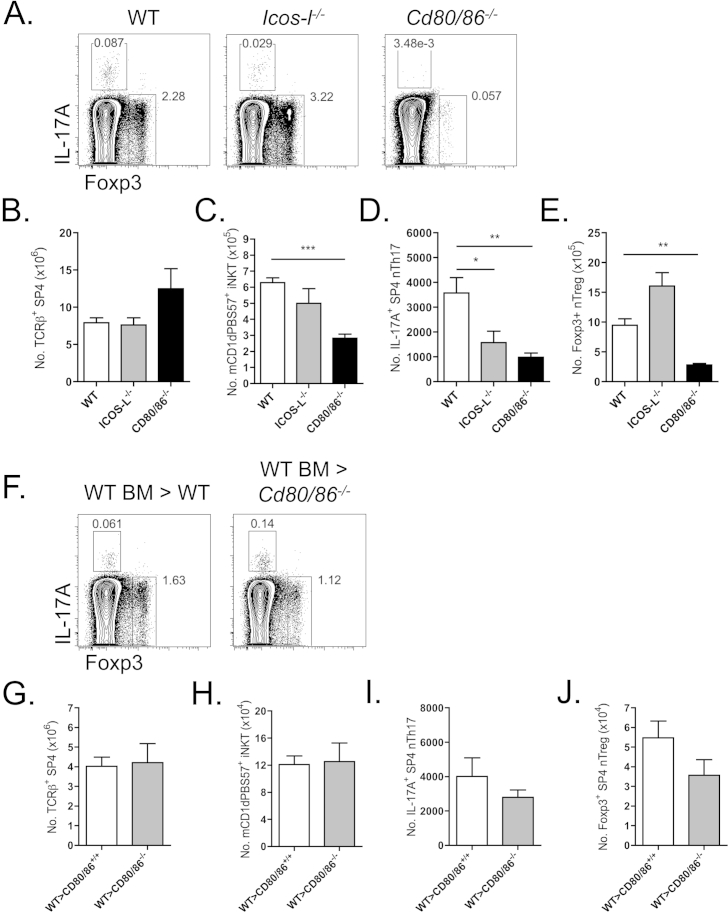
**ICOS-ligand and CD80/86 co-stimulation impacts upon nTh17 development**. (A) Representative flow cytometric staining of PMA/Ionomycin stimulated thymocytes isolated from WT, *ICOS-L*^−/−^ and *CD80/86*^−/−^ adult murine thymus. Representative plots gated on TCRβ^+^mCD1d-PBS57^−^CD4^+^CD8^−^TCRγδ^−^ cells. (B) Absolute numbers of TCRβ^+^mCD1d-PBS57^−^CD4^+^CD8^−^ SP4 thymocytes, (C) mCD1d-PBS57^+^ iNKT cells, (D) TCRβ^+^mCD1d-PBS57^−^CD4^+^CD8^−^TCRγδ^−^IL-17A^+^ nTh17 cells and (E) TCRβ^+^mCD1d-PBS57^−^CD4^+^CD8^−^TCRγδ^−^Foxp3^+^ nTreg isolated from WT (open bars), *ICOS-L*^−/−^ (grey bars) and *CD80/86*^−/−^ (black bars) mice. Data is from a minimum of 3 independent experiments, with n ≥ 8 mice per group. Data statistically analyzed using a non-parametric Kruskal–Wallis one-way analysis of variance with Dunn's post-test. (F) Representative FACs plots of thymocytes isolated from lethally irradiated CD45.2^+^ WT or CD45.2^+^*CD80/86*^−/−^ hosts reconstituted with congenic CD45.1^+^ CD3e^+^-depleted bone marrow, plots gated as in (A). Absolute numbers of (G) TCRβ^+^mCD1d-PBS57^−^CD4^+^CD8^−^ SP4 thymocytes, (H) mCD1d-PBS57^+^ iNKT cells, (I) TCRβ^+^mCD1d-PBS57^−^CD4^+^CD8^−^TCRγδ^−^IL-17A^+^ nTh17 cells and (J) TCRβ^+^mCD1d-PBS57^−^CD4^+^CD8^−^TCRγδ^−^Foxp3^+^ nTreg from WT bone marrow (BM) transferred into WT hosts (open bars) and WT BM into *CD80/86*^−/−^ hosts (grey bars). Error bars represent SEM. Data analyzed using a Mann Whitney U test, all data non-significant. Data is from 3 independent experiments with n ≥ 5 mice per group.

Thymic nTh17 constitutively express CD28 superfamily members, including ICOS, within thymic microenvironments [8]. Due to the critical reliance of nTh17 upon MHC class II (Fig. 3B), and proposed selection based upon strong TCR-affinity for self-antigen [8], the supplementary role of costimulation provided by ICOS-ligand and CD80/86 ligands that are constitutively expressed by thymic APC subsets, was investigated (Fig. 5). Flow cytometric analysis of αβT-cell development in WT, ICOS-L^−/−^ and CD80/86^−/−^ mice revealed normal development of conventional TCRβ^+^ SP4 thymocytes in all mice (Fig. 5B). However, analysis of natural αβT-cells revealed a selective reduction of nTh17 only in ICOS-L-deficient mice, whereas both nTreg and iNKT numbers were unaffected. In contrast, CD80/86-deficient mice exhibited significantly reduced numbers in all three nTh17, nTreg and iNKT subsets (Fig. 5A, C–E).

With the most significant decrease in nTh17 having being observed in the absence of CD80/86 costimulation (Fig. 5D), we investigated whether the provision of CD80/86 by radio-resistant thymic stromal elements, presumably mTEC, accounted for the dependency of nTh17 upon functionally intact medullary compartments. T-cell-depleted WT CD45.1^+^ bone marrow cells were transferred into lethally irradiated CD45.2^+^ WT or CD45.2^+^ CD80/86^−/−^ hosts and the presence of CD45.1^+^ donor-derived natural αβT-cell subsets determined by flow cytometry (Fig. 5F). As demonstrated in Fig. 5G–J, iNKT, nTreg and nTh17 were all found to be present at normal numbers in CD80/86^−/−^ hosts receiving WT bone marrow. Together these date demonstrate that although costimulation provision via B7 family members including CD80/86 and ICOS-L is important for normal nTh17 development, the selective absence of CD80/86 from otherwise functionally intact medullary thymic stromal compartments is dispensable for the development of such cells, presumably due to the provision of costimulation by BM-derived cells including thymic DC.

### 3.4 Thymic nTh17 homeostasis is independent of constitutive mTEC-derived inducible nitric oxide synthase

**Fig. 6:**
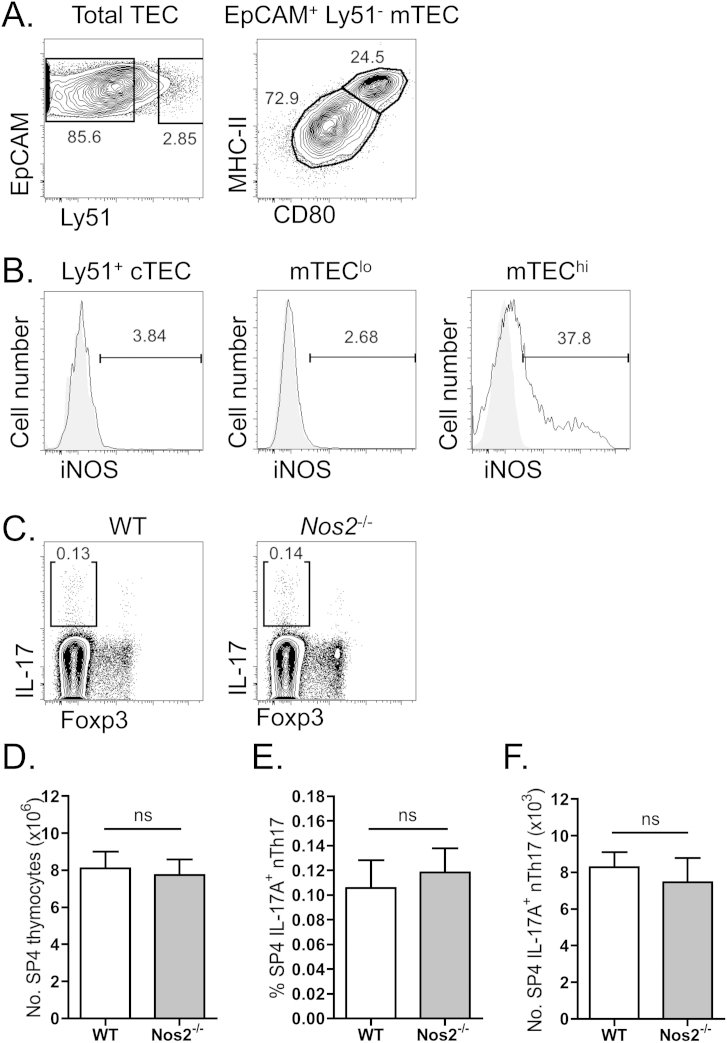
**Constitutive expression of inducible nitric oxide synthase by mTEC is not essential for thymic nTh17 regulation**. (A) Representative flow cytometry plots for the discrimination of EpCAM^+^ Ly51^+^ cortical thymic epithelial cells (cTEC), EpCAM^+^Ly51^−^MHCII^lo^CD80^lo^ medullary thymic epithelial cells (mTEC^lo^) and EpCAM^+^Ly51^−^MHCII^hi^CD80^hi^ (mTEC^hi^). Plots gated on CD45^−^ cells. (B) Representative FACs staining of iNOS expression in cTEC, mTEC^lo^ and mTEC^hi^ as gated in (A), open histogram iNOS antibody staining on WT adult TEC, grey filled histogram control iNOS staining on *Nos2*^−/−^ adult TEC. Data representative of 3 independent experiments. (C) Representative FACs staining of IL-17^+^ nTh17 and Foxp3^+^ nTreg in either WT or *Nos2*^−/−^ adult mice. Plots gated on TCRβ^+^mCD1d-PBS57^−^CD4^+^CD8^−^TCRγδ^−^ cells. (D) Absolute numbers of TCRβ^+^mCD1d-PBS57^−^CD4^+^CD8^−^ SP4 thymocytes, and the frequency (E) and absolute number (F) of TCRβ^+^mCD1d-PBS57^−^CD4^+^CD8^−^TCRγδ^−^IL-17A^+^ nTh17 cells are depicted. WT (open bars) and *Nos2*^−/−^ (grey bars). Error bars represent SEM. Using a Mann Whitney U test, ns (not significant). Data representative of 3 independent experiments, n ≥ 6 mice per group.

The dysregulated development and/or activity of Th17 cells has been linked to the manifestation of inflammatory pathology. We therefore sought to investigate whether, in addition to potential positive regulation of nTh17 development, the thymic medulla utilizes similar mechanisms to that reported for the negative regulation of inducible Th17 to constrain nTh17 cells. Given recent reports that inducible nitric oxide synthase (iNOS) plays a pivotal role in restricting inducible Th17 cells, we investigated the expression of iNOS within thymic microenvironments. Critically, flow cytometric analysis of iNOS expression revealed that following discrimination of cTEC (EpCAM^+^Ly51^+^), mTEC^lo^ (EpCAM^+^Ly51^−^CD80^lo^MHCII^lo^) and mTEC^hi^ (EpCAM^+^Ly51^−^CD80^hi^MHCII^hi^) fractions of thymic epithelium (Fig. 6A), constitutive expression of iNOS was selectively detected within the mTEC^hi^ population of adult steady-state murine thymus (Fig. 6B). Given that intrathymic iNOS expression correlated with thymic medullary compartments, including the mTEC^hi^ fraction that encompasses Aire^+^ mTEC [38], that we show here critically regulate nTh17 cells (Fig. 4), the frequency and number of nTh17 cells was analyzed within iNOS-deficient mice (Fig. 6C–F). Notably, no perturbation in either total CD4^+^8^−^ SP4 thymocyte numbers, nor the percentage or absolute numbers of nTh17 was observed. Together these data provide novel insights into the constitutive expression of iNOS specifically within mTEC^hi^ compartments, and further reveal that in direct contrast to inducible Th17, iNOS is not a critical factor regulating the differentiation of thymic nTh17 cells.

## 4 Discussion

Thymic natural Th17 cells have recently emerged as a novel CD4^+^ T-cell subset that can arise following intrathymic priming, and may contribute to either protective or pathogenic inflammatory responses [39]. The natural capacity of nTh17 to rapidly produce pro-inflammatory IL-17 cytokine has recently generated interest in this newly identified T-cell subset, although the precise functional contribution of such cells has remained enigmatic. Whilst the innate-like production of IL-17 by nTh17 has lead to descriptions of an important role for such cells in early protective responses to immunological challenge at mucosal surfaces [10], the potential of IL-17 production by innate-like cells to drive autoimmune pathology [40], implies that strict developmental mechanisms may be required to regulate the development and activity of nTh17 to reduce the risk of autoimmunity. In this regard, nTh17 have recently been identified as a cellular source of IL-17 in an experimental mouse psoriasis-like disease model [9], and further, the aberrant development of increased nTh17 numbers has been indirectly linked to the manifestation of autoimmune disease [12]. These data would imply that tight control of nTh17 cells is required to counterbalance any positive immunoprotective activity with their potential to exacerbate autoimmune disease. Despite this, the mechanisms controlling their intrathymic development are currently unclear [16]. Given this uncertainty, we examined the requirements for nTh17 cell development in relation to natural αβT-cell subsets. Here, we show that the thymus medulla is essential for the intra-thymic generation of nTh17, via mechanisms involving Aire and the costimulatory molecules ICOS-L and CD80/86. Further, we show that in contrast to the reported role of iNOS in the negative regulation of conventional Th17, despite constitutive expression by mTEC, thymic nTh17 are unaffected by an absence of iNOS. Collectively, our findings identify differing roles for thymic medullary microenvironments in the generation of natural CD4^+^ αβT-cell subsets and reveal that nTh17 development and conventional Th17 differentiation are differentially regulated.

Although the thymic medulla is required for the regulation of heterogeneous natural CD4^+^ T-cells, this occurs via several differential mechanisms. In the case of iNKT, mTEC are partially required for their provision of IL-15/IL-15Rα following CD1d-dependent selection on CD4^+^8^+^ thymocytes, whereas nTreg are at least in part dependent upon mTEC-derived self-antigen presentation for their selection [33,41]. Interestingly, in contrast to the dependence of iNKT upon mTEC-dependent provision of IL-15, recent experiments demonstrated that thymic nTh17 are conversely negatively regulated by IL-15, being present at elevated numbers in mice lacking IL-15 production by radio-resistant thymic epithelial compartments and correlating with autoimmune pathology [12]. Additional studies have further described a positive role for IL-15 in the maturation of Foxp3^+^CD25^−^ nTreg precursors [42]. Given the opposing action of IL-15 on nTh17 and Foxp3^+^CD25^−^ nTreg precursor development, and in light of experiments demonstrating the plasticity between Treg and Th17 lineages, including IL-17 upregulation in T-cells skewed to but unable to complete Treg lineage differentiation, the direct developmental relationship and regulation of nTreg and nTh17 are of potential importance in terms of the thymic medullas role in the controlling the balance of either protective or inflammatory T-cell development.

In contrast to iNKT, nTreg appear to share similar thymic selection mechanisms with nTh17. For example, our data revealing the mTEC dependency of nTh17 parallels that reported for nTreg development, whereby the selective expression of MHC class II by functionally intact cTEC in RelB-deficient thymus grafts mediates conventional SP4 thymocyte positive selection but is unable to lead to the intrathymic priming of both nTh17 and nTreg [3]. The selection of both nTreg and nTh17 is reportedly increased in double transgenic mice expressing MHC class II-restricted TCRs and specific cognate peptide within thymic microenvironments, supporting the idea that both cell types demonstrate a self-reactive bias [8,43]. This fits well with data presented here indicating a reduction of nTh17 in Aire-deficient mice, which may result from alterations in the array of self-antigens normally expressed by mTEC. Interestingly, these data align with our results presented here, and previous studies demonstrating a significant reduction of the proportion of nTreg in Aire knockout mice [35–37], and lie in contrast to normal iNKT development reported to occur in Aire-deficient thymic microenvironments [44]. Whether subtle changes in Aire expression regulate the development of nTh17 cells in a dose-dependent manner, akin to that described for the negative selection of autoreactive thymocytes [45], remains an open question and will provide an interesting area of investigation in future experiments examining the intrathymic regulation of nTh17 development. Of note, although one study has examined the generation of nTh17 in double TCR transgenic and thymic neo-self-antigen transgenic mice, expression of agonist neo-antigen within thymic microenvironments was restricted to thymic epithelium, but did not discriminate between a role for either cTEC or mTEC presentation of cognate peptide [8,46]. It therefore remained unclear whether nTh17 could be selected solely via interactions with cTEC or whether mTEC were required for the skewing of positively selected CD4^+^ αβTCR thymocytes towards the nTh17 lineage. Through the analysis of nTh17 development in RelB-deficient thymus grafts, we here provide evidence that despite normal positive selection and subsequent maturation of conventional SP4 thymocytes [3], selection of nTh17 cells requires mTEC-dependent mechanisms, thereby demonstrating that MHC class-II expression by cTEC is insufficient to drive nTh17 selection.

Interestingly, the use of bone marrow chimeras to specifically target loss of CD80/86 expression to mTEC suggests that radiosensitive cells, most likely thymic DC, are able to act as a source of CD80/86-mediated costimulation for normal natural αβT-cell development, at least in numerical terms. It is therefore conceivable that the role of mTEC in the development of nTh17 may be to indirectly act as a local reservoir of self-antigen subsequently acquired and cross-presented by thymic DC [47–49]. Consistent with a direct capacity of DC to drive natural αβT-cell development, studies have revealed that targeted expression of agonist antigen to CD11c^+^ DC is capable of directly driving antigen-specific thymic nTreg development [50]. It is currently unclear at which stage co-stimulation is required during nTh17 development in the thymus, specifically in regards to either initial selection/specification or post-election expansion. However, given that nTreg demonstrate a two-step reliance upon TCR and costimulation followed by a cytokine-dependent phase driven by common gamma chain-dependent cytokines IL-2/IL-15 [51,52], it could be speculated that nTh17 demonstrate similar developmental regulation. As yet, the precise cytokine regulation of nTh17 is uncertain, although in contrast to inducible Th17 some experimental evidence may suggest that nTh17 development in a polyclonal repertoire is IL-6 independent [8,9]. In combination with our data reported here regarding the contrasting role of iNOS in the regulation of nTh17, these studies support the proposal that nTh17 and inducible Th17 have differential signaling requirements.

In summary, this study demonstrates an essential role for the thymus medulla in the regulation of intrathymic nTh17 cell development. The medullary regulation of nTh17 encompasses mTEC Aire-dependent regulation of nTh17 and provision of costimulation via both ICOS-L and CD80/86 pathways, but is independent of iNOS-mediated suppression. These data highlight the distinct medullary-dependent maturational requirements of natural αβT-cell subsets, reveal novel differential regulation of nTh17 compared to conventional Th17 cells and provide a basis for further studies investigating the development of immunoregulatory natural T-cell subsets associated with modulation of inflammatory and autoimmune responses.

## Competing interest

The authors have no competing financial interests.
