# Supplementary material for: Natural Th17 cells are critically regulated by functional medullary thymic microenvironments
Source: J Autoimmun. 2015 Sep;63:13–22. doi: 10.1016/j.jaut.2015.06.008 (PMC4570931; doi:10.1016/j.jaut.2015.06.008)
Supplement: Supplementary file 1 [file mmc1.pdf]

Supplementary Figure 1.

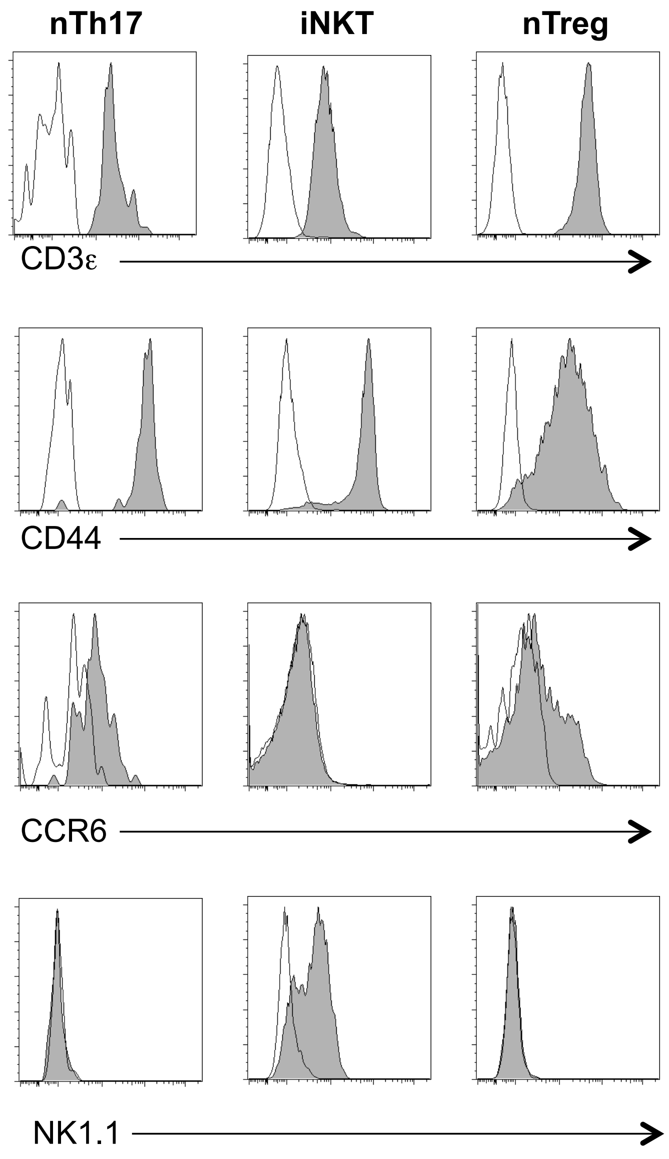

Supplementary Figure 1. Comparative analysis of the cell surface phenotype of intrathymic natural αβT-cell subsets.

Flow cytometric analysis of thymic nTh17, iNKT and nTreg following isolation of adult wildtype murine thymocytes and *in vitro* stimulation with PMA and Ionomycin, in the presence of Brefeldin A. nTh17 cells gated on $TCR\beta^{+}mCD1d\text{-}PBS57^{-}CD4^{+}CD8^{-}TCR\gamma\delta^{-}Foxp3^{-}IL\text{-}17^{+}$ cells, iNKT cells gated on $TCR\beta^{+}mCD1d\text{-}PBS57^{+}$ cells, and nTreg gated on on $TCR\beta^{+}mCD1d\text{-}PBS57^{-}CD4^{+}CD8^{-}TCR\gamma\delta^{-}Foxp3^{+}$ cells. Histogram plots depict staining of the stated cell surface protein (grey filled line), isotype staining controls represented by black open lines. Data representative of two independent experiments.
